# Structure-Function Dissection of *Myxococcus xanthus* CarD N-Terminal Domain, a Defining Member of the CarD_CdnL_TRCF Family of RNA Polymerase Interacting Proteins

**DOI:** 10.1371/journal.pone.0121322

**Published:** 2015-03-26

**Authors:** Diego Bernal-Bernal, Aránzazu Gallego-García, Gema García-Martínez, Francisco García-Heras, María Angeles Jiménez, S. Padmanabhan, Montserrat Elías-Arnanz

**Affiliations:** 1 Departamento de Genética y Microbiología, Área de Genética (Unidad Asociada al IQFR-CSIC), Facultad de Biología, Universidad de Murcia, Murcia, Spain; 2 Instituto de Química Física ‘Rocasolano’, Consejo Superior de Investigaciones Científicas (IQFR-CSIC), Serrano, Madrid, Spain; MRC National Institute for Medical Research, UNITED KINGDOM

## Abstract

Two prototypes of the large CarD_CdnL_TRCF family of bacterial RNA polymerase (RNAP)-binding proteins, *Myxococcus xanthus* CarD and CdnL, have distinct functions whose molecular basis remain elusive. CarD, a global regulator linked to the action of several extracytoplasmic function (ECF) σ-factors, binds to the RNAP β subunit (RNAP-β) and to protein CarG via an N-terminal domain, CarDNt, and to DNA via an intrinsically unfolded C-terminal domain resembling eukaryotic high-mobility-group A (HMGA) proteins. CdnL, a CarDNt-like protein that is essential for cell viability, is implicated in σ^A^-dependent rRNA promoter activation and interacts with RNAP-β but not with CarG. While the HMGA-like domain of CarD by itself is inactive, we find that CarDNt has low but observable ability to activate ECF σ-dependent promoters *in vivo*, indicating that the C-terminal DNA-binding domain is required to maximize activity. Our structure-function dissection of CarDNt reveals an N-terminal, five-stranded β -sheet Tudor-like domain, CarD_1–72_, whose structure and contacts with RNAP-β mimic those of CdnL. Intriguingly, and in marked contrast to CdnL, CarD mutations that disrupt its interaction with RNAP-β did not annul activity. Our data suggest that the CarDNt C-terminal segment, CarD_61–179_, may be structurally distinct from its CdnL counterpart, and that it houses at least two distinct and crucial function determinants: (a) CarG-binding, which is specific to CarD; and (b) a basic residue stretch, which is also conserved and functionally required in CdnL. This study highlights the evolution of shared and divergent interactions in similar protein modules that enable the distinct activities of two related members of a functionally important and widespread bacterial protein family.

## Introduction

In the Gram-negative soil bacterium *Myxococcus xanthus*, a 316-residue global regulatory protein, CarD, is involved in the control of light-induced carotenogenesis, starvation-induced development of multicellular fruiting bodies, and other processes [1,2]. In the response to light, CarD is required for the activation of P_QRS_, a promoter that depends on the alternative extracytoplasmic function σ (ECF-σ) factor CarQ [3]. At least another ten of the ~45 ECF-σ factors in *M*. *xanthus* also depend on CarD [4], which has a striking domain architecture. Its ~140-residue intrinsically unfolded C-terminal DNA-binding domain resembles eukaryotic high-mobility group A (HMGA) proteins and is composed of an acidic segment followed by a region containing the hallmark basic RGRP or AT-hook repeats [5–7]. The CarD N-terminal domain, CarDNt (~180 residues), has defined tertiary structure and interacts with CarG (a 170-residue zinc-associated protein required in all CarD-dependent processes) and with the RNA polymerase (RNAP) β subunit, but not with DNA [4,5,8–10].

CarD, CarG, and their orthologs have been found only in *M*. *xanthus* and related myxobacteria thus far [2,5,8]. However, CarDNt is a defining member of the large CarD_CdnL_TRCF family of bacterial RNAP-interacting proteins (PF02559 in the protein family database, http://pfam.sanger.ac.uk), which includes two other classes of proteins widely distributed in bacteria [7,9,11,12]). One class comprises the transcription-repair coupling factors (TRCF), large and conserved multidomain proteins involved in the repair of DNA lesions encountered by the transcribing complex [13–16], whose RNAP-interacting domain (TRCF-RID) shares sequence similarity with CarDNt [7]. The second class includes standalone proteins with sizes between 160 and 200 residues, similar in sequence to CarDNt but lacking the characteristic HMGA-like domain of CarD [9]. We named these CdnL (for CarD N-terminal like) to distinguish them from CarD, and because both proteins coexist in *M*. *xanthus* [11]. We have shown that CarDNt and CdnL cannot be functionally interchanged *in vivo*, and while CarD can be deleted in *M*. *xanthus* without impairing viability, CdnL is essential for cell growth and survival [11]. CdnL is also an essential factor in the few other bacterial species where it has been studied, such as the pathogens *Mycobacterium tuberculosis* and *Borrelia burgdorferi*, both of which lack CarD [12,17]. CdnL interacts with RNAP and has been linked to rRNA promoters, where it stabilizes the formation of transcriptionally competent open complexes (RP_o_) by RNAP containing the major housekeeping σ, σ^A^ [10,12,18,19]. Thus, whereas CarD is linked to the action of various ECF-σ factors at their target promoters, CdnL has been implicated in σ^A^-dependent rRNA promoter activation. The molecular basis for these distinct modes of action remains to be elucidated, and insights into this can come from structure-function analysis of CarD and CdnL. High-resolution structural data and structure-based functional analyses have been reported for CdnL [10] and its homologs in mycobacteria and *Thermus thermophilus* ([18,20–22]. The present study reports our findings with CarDNt, the N-terminal domain of CarD, and counterpart of full-length CdnL.

We find that CarDNt exhibits low but observable activity *in vivo*, while the C-terminal HMGA-like DNA-binding domain of CarD is inactive on its own. RNAP-β recognition by CarDNt is mediated by its N-terminal 72-residue module, CarD_1–72_, whose solution structure determined by NMR and contacts inferred by structure-directed mutagenesis closely match those observed for CdnL. However, whereas disrupting the interaction of CdnL with RNAP-β caused a severe loss of function and impaired cell growth and survival, equivalent mutations in CarD or CarDNt did not drastically diminish its activity. We also found that the CarDNt stretch spanning residues 61 to 179 (CarD_61–179_), which is not involved in the interaction with RNAP-β, mediates at least two functionally critical activities: interaction with CarG, and an undefined activity provided by a stretch of basic residues that does not participate in the interaction with CarG. The equivalent domain of CdnL, which is also indispensable for its distinct role, conserves the functionally crucial basic residue segment but not the interaction with CarG. Our data reveal structural modules with shared and divergent roles in CarD and CdnL that have evolved to enable their distinct functions in *M*. *xanthus*, and will be useful for understanding the structure-function relationships underlying the enigmatic modes of action of this widely distributed class of bacterial RNAP-interacting proteins.

## Materials and Methods

### Strains, plasmids, and growth conditions

Table A in S1 File lists strains and plasmids used in this study. *M*. *xanthus* vegetative growth was carried out at 33°C in CTT (casitone Tris) medium supplemented as required with antibiotic (40 μg/ml kanamycin, Km). For light-induced carotenogenesis, liquid cultures of the *M*. *xanthus* strain were grown to exponential phase (optical density at 550 nm, OD_550_, of ~0.8) in CTT from which 5 μl drops were spotted on two CTT plates. After a ~12 h incubation in the dark at 33°C, one plate continued to be in the dark while the other was exposed to light (three 18-W fluorescent lamps at ~10 W/m^2^ intensity). For specific β-galactosidase activity (β-Gal activity) measurements (see below), liquid cultures grown in CTT to OD_550_ of ~0.4 in the dark were divided into two and one was grown in the dark and the other in the light. *E*. *coli* strains used were DH5α [23] for plasmid constructs, BL21(DE3) [24] for protein overexpression, and BTH101 (*cya* ^−^) [25] for two-hybrid analysis. These were grown in Luria-Bertani (LB) broth at 37°C. Proteins were overexpressed at 18°C overnight with 0.5 mM IPTG (isopropyl β-D-1-thiogalactopyranoside) in LB or, for [^13^C, ^15^N]-labeled proteins, in MOPS minimal medium containing 2.5 g/l ^13^C_6_-glucose and 1 g/l ^15^NH_4_Cl as the sole carbon and nitrogen sources [20,26,27].

Standard protocols and kits were used for plasmid constructs, all of which were verified by DNA sequencing. Site-directed *carD* mutants were obtained by overlapping PCR or as synthetic genes (GenScript). Plasmid pMR2603, with a Km^R^ marker for positive selection and *galK* for galactose sensitivity (Gal^S^) negative selection and used in complementation analysis, has been described previously [9]. It has an EcoRI site into which *carD* or its given variant can be introduced flanked at the 5´ and 3´ ends, respectively, by ~1 kb of the DNA upstream and ~2 kb downstream of *carD* in the genome for plasmid integration into the chromosome by homologous recombination. The construct with a *carD* allele was electroporated into the Δ*carD* strain MR1900, resulting in Km^R^ Gal^S^ merodiploids. Recovery of the Car^+^ color phenotype for light-induced carotenogenesis (yellow in the dark; red in the light) indicated complementation. To generate haploid strains bearing the desired allele, the Km^R^ Gal^S^ merodiploids were grown without antibiotic for several generations and plated on CTT containing 10 mg/ml galactose to select for plasmid excision via intramolecular recombination events. Km^S^ Gal^R^ haploids that retained the desired *carD* allele were identified by PCR and checked by DNA sequencing, with stable protein expression verified in immunoblots of whole cell extracts, as detailed below. These were then electroporated with pDAH217 (Km^R^) bearing a reporter *lacZ* transcriptional probe fused to the *carD*-dependent light-inducible P_QRS_ promoter [28], whose activity in the dark or under light was assessed quantitatively by β-Gal activity measurements, as described below. Since DdvS function is linked to DdvA inactivation, CarD-dependent P_*ddvS*_ promoter activity was assessed in a Δ*ddvA* strain with intact *carD*, or with the Δ*carD*, *carDNt* or Δ*carDNt* alleles (Table A in S1 File). The first two strains were obtained previously [4]. The remaining two were generated by inserting the *carDNt* or Δ*carDNt* allele into the EcoRI site of pMR2603, and then electroporating each plasmid into the Δ*ddvA* Δ*carD* strain (Table A in S1 File; [4]), followed by isolation of haploid strains with the *carDNt* or Δ*carDNt* allele using procedures described above. The P_*ddvS*_-lacZ reporter probe [4] was then electroporated into each of these strains for β-Gal activity measurements.

### Protein purification and analysis

CarDNt and CarD_1–72_ were overexpressed as intein fusions and purified using chitin resin with the intein tag removed by on-column intramolecular cleavage in the presence of 50 mM dithiothreitol using the IMPACT kit and protocols (New England Biolabs). The cleaved protein was passed again through a small amount of chitin resin to eliminate residual intein and dialyzed extensively against 100 mM NaCl, 50 mM phosphate buffer, pH 7.0, and 0.05% NaN_3_, and concentrated using Amicon Ultra (molecular weight cut-off 3,000 Da) [5,20]. Protein identities were verified using N-terminal sequencing and mass spectrometry (which confirmed the presence of the additional non-native N-terminal AGH that remains after intein tag removal), and concentrations were estimated using the BioRad protein assay kit.

### Immunoblot analysis

Immunoblot (Western) analysis of whole cell extracts was carried out as described previously [5,11]. Briefly, each strain was grown in 10 ml CTT at 33°C to an OD_550_ of ~0.7, and cells from 500 μl were pelleted by centrifugation and stored at −70°C until required. The cell pellets were thawed, resuspended in 300 μl buffer (100 mM NaCl, 50 mM Tris-HCl pH 7.5, 2 mM EDTA, 1 mM each of phenylmethyl sulfonyl fluoride and benzamidine, 30 μl protease inhibitor mixture (Sigma), and 20 μl of each sample were loaded into a 4–12% Bis-Tris Criterion XT (BioRad) precast gel and subjected to SDS/PAGE electrophoresis. Proteins were transferred to a PVDF membrane using a semi-dry electroblotting unit, and analyzed using the ECL kit (GE Healthcare Life Sciences), anti-CarD monoclonal antibodies [5,7] and, as loading control, polyclonal anti-CdnL antibodies [11].

### **Bacterial two-hybrid (BACTH) analysis and** β-galactosidase activity

The *E*. *coli* BACTH system used is based on functional complementation of the T25 and T18 fragments of the *Bordetella pertussis* adenylate cyclase catalytic domain when two test proteins interact [25]. Coding regions of interest were PCR-amplified and cloned into the XbaI and BamHI sites of pKT25, pUT18 or pUT18C (Table A in S1 File). Given pKT25/pUT18 or pUT18C pairs were electroporated into *E*. *coli* BTH101 (*cya* ^-^), a pair with vector alone serving as negative control. Interaction was assessed qualitatively from the blue color developed on X-Gal plates or quantitatively from β-Gal activity (in nmol of *o*-nitrophenyl β-D-galactoside hydrolysed/min/mg protein, from the mean and standard error of three or more independent experiments) measured for liquid cultures in a SpectraMax 340 microtitre plate reader (Molecular Devices) as described elsewhere [29].

### Circular dichroism (CD) spectroscopy

Far-UV CD spectra were recorded in a Pistar unit (Applied Photophysics, UK) calibrated with -10-camphorsulfonic acid and coupled to a Peltier temperature control unit/Neslab RTE-70 water bath. Data were collected in 0.2 nm steps in the adaptive sampling mode at 25°C with 5–10 μM protein, 100 mM KF, 7.5 mM phosphate buffer (pH 7.5), 1 mm path length, 2 nm slit width, and averaged over three scans. Helix contents were estimated from [Θ]_222_, the mean residue ellipticity at 222 nm in degcm^2^dmol^−1^ using [Θ]_222_ = 895 for 0% helix and (−37,750)(1–3/N_r_) for 100% helix, where N_r_ is the number of residues [30].

### NMR

NMR data were acquired in a Bruker AV-600 or AV-800 US2 spectrometer equipped with a z-gradient triple resonance cryoprobe using 0.5 or 0.2 mL samples of 0.5–1 mM protein in 100 mM NaCl/50 mM sodium phosphate buffer (pH 7.0, calibrated with a glass microelectrode and uncorrected for isotope effects)/0.05% NaN_3_ in 9:1 v/v H_2_O/D_2_O or pure D_2_O. Probe temperatures were set using a methanol sample. Standard triple resonance NMR methods were used for data acquisition, processing, and ^1^H/^15^N/^13^C NMR chemical shift assignments, which were deposited at BioMagResBank (http://www.bmrb.wisc.edu/; accession code: 18194). Distance constraints, obtained from a 3D NOESY [^1^H-^13^C]-HSQC and two 2D [^1^H-^1^H]-NOESY spectra (mixing times of 80 ms and 150 ms, respectively) in H_2_O/D_2_O and/or D_2_O, and (ϕ, ψ) torsion angle constraints, from TALOS, were used as input in structure calculations using a standard iterative protocol of the program CYANA 2.1 [20,31,32]. Of the 100 conformers generated, 20 with the lowest target function values were energy minimized using AMBER9 (Case DA, Darden TA, Cheatham III TE, University of California, San Francisco, 2006). The quality of the final structures was assessed with PROCHECK/NMR [33]. MOLMOL [34] was used for representations and analysis of the calculated structures, whose coordinates have been deposited in the Protein Data Bank (accession code: 2LT1).

## Results

### CarDNt can partially complement the lack of CarD

**Fig 1:**
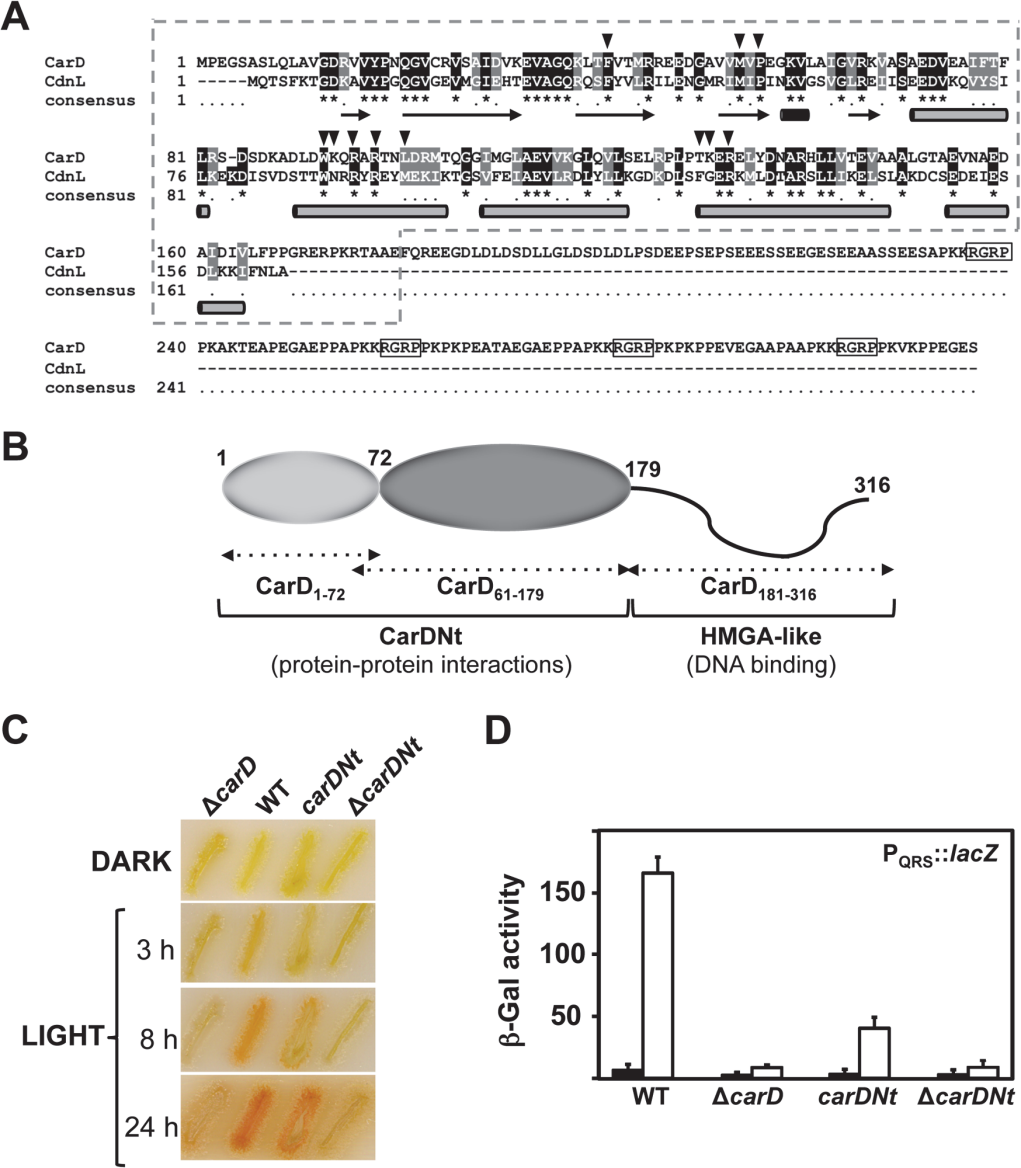
CarD domains and interactions. (**A**) Sequence alignment of *M*. *xanthus* CarD and CdnL (NCBI accession codes CAA91224 and YP_630846, respectively). CarDNt corresponds to the 179-residue N-terminal CarD segment enclosed by the dashed line, and the remaining C-terminal segment to the HMGA-like region (with the four RGRP AT-hooks boxed). Arrowheads point to CarDNt residues analyzed by site-directed mutagenesis in this study. Below the sequence, arrows correspond to β-strands, the short black rod to a 3_10_ helix and the grey rods to α-helices in the NMR solution structure determined for CdnL [10]. (**B**) Schematic showing CarD domain architecture. The two modules involved in distinct protein-protein interactions in the structured CarDNt domain are shown as ellipsoids shaded in light and dark grey. The intrinsically unstructured C-terminal HMGA-like DNA binding domain is depicted as a wavy line. Numbers indicate residues delimiting each CarD module. (**C**) Color phenotype of *M*. *xanthus* strains with wild-type *carD* (WT), Δ*carD*, *carDNt*, and Δ*carDNt* alleles streaked on CTT plates and grown in the dark and then in the light for the times indicated. (**D**) Reporter P_QRS_::*lacZ* expression (β-Gal activity) measurements for exponentially growing cells of each of the indicated strains in the dark (filled bars) or after 14 h in the light (unfilled bars). The mean and standard error of three independent experiments are shown.

The sequence alignment in Fig. 1A illustrates the similarity between CarDNt and CdnL, the presence of the additional HMGA-like C-terminal region in CarD, and secondary structure elements in CdnL based on its NMR solution structure [10]. We previously showed that deleting the CarDNt module leads to loss of CarD activity *in vivo* [9]. Whether CarDNt on its own had activity *in vivo* remained to be tested and was examined here. In the *M*. *xanthus* response to light, CarD is implicated in the activation of the *carQRS* regulatory operon from its promoter, P_QRS_, which is recognized by RNAP holoenzyme containing the ECF-σ factor CarQ [1,2]. As a consequence, wild-type cells are yellow in the dark but turn reddish in the light due to carotenoid production (Car^+^ phenotype; Fig. 1C). On the other hand, a *carD*-deleted (Δ*carD)* strain does not become red in the light (Car^-^ phenotype; Fig. 1C). The strain expressing only the CarD C-terminal HMGA-like domain (Δ*carDNt* allele) exhibited a Car^-^ phenotype (Fig. 1C; [9]), indicating the essential role of CarDNt in CarD function. To test the effect *in vivo* of deleting the HMGA-like domain, we constructed a strain with the native *carD* allele replaced by one that encodes CarDNt (*carDNt* allele). In marked contrast to the Δ*carD* or Δ*carDNt* strains, the strain expressing *carDNt* acquired the red color associated with carotenogenesis, albeit at a slower rate than the wild-type strain (Fig. 1C). Consistent with the color phenotypes, the light-inducible P_QRS_-*lacZ* reporter activity in the *carDNt* strain was about a fourth of that observed in the wild type, but significantly higher than the very low basal levels observed in the Δ*carD* or Δ*carDNt* strains (Fig. 1D). Hence, light-induced carotenogenesis, which is severely impaired on deleting the entire *carD* or only the part encoding CarDNt, can be partially restored by CarDNt.

To further corroborate this partial activity of CarDNt *in vivo* and that it is not specific to P_QRS_, we examined the effect of the above domain truncations on expression from the CarD-dependent promoter, P_*ddvS*_, unrelated to the light response. This promoter also requires an ECF-σ factor, DdvS, which becomes active when its cognate anti-σ DdvA is inactivated [4]. Since the specific external signal that triggers DdvA inactivation is unknown, reporter P_*ddvS*_-*lacZ* activity in the presence of the different *carD* alleles was determined in a Δ*ddvA* genetic background. Again, in the corresponding *carDNt* strain, reporter *lac*Z activity was about 4-fold lower than in the strain with the wild-type *carD*, but significantly higher than the negligible levels observed in the Δ*carD* or Δ*carDNt* strains (Fig. A in S1 File). Thus, while the HMGA-like C-terminal domain is necessary to achieve maximal CarD activity, CarDNt is absolutely essential for CarD function and by itself exhibits low-level activity relative to the full-length protein.

### Modular dissection of CarDNt interactions with RNAP-β and CarG

**Fig 2:**
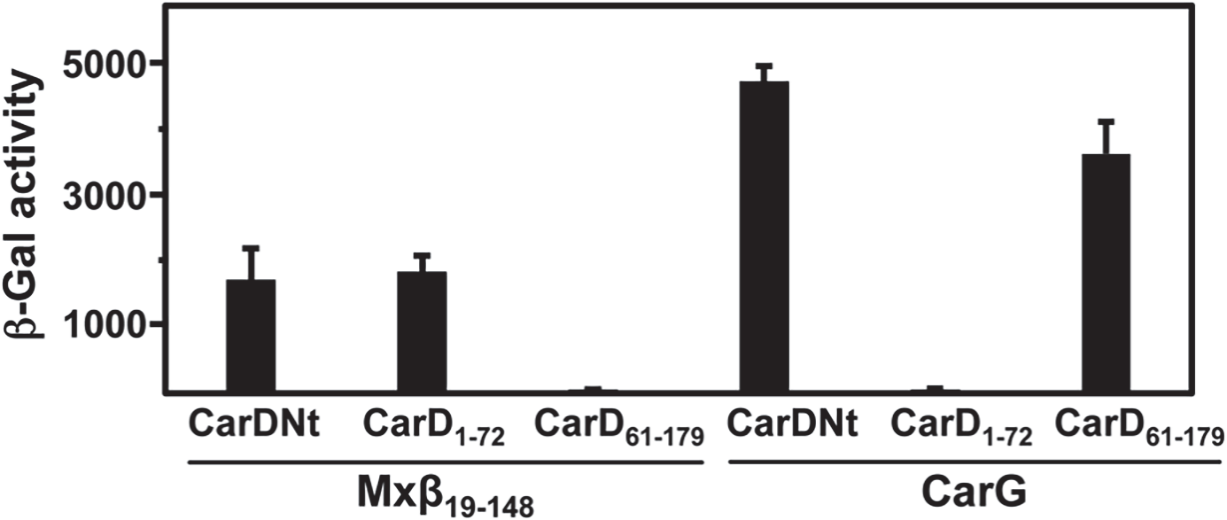
Modular dissection of CarDNt interactions with RNAP and CarG. BACTH analysis showing reporter *lacZ* expression (β-Gal activity) in *E*. *coli* transformed with pKT25-derived plasmids expressing CarDNt, CarD_1–72_ or CarD_61–179_ and with pUT18C constructs expressing Mxβ_19–148_ or CarG. The mean and standard error of three independent experiments are shown.

Both CarDNt and CdnL interact with the *M*. *xanthus* RNAP-β fragment spanning residues 19 to 148, Mxβ_19–148_ [11]. In CdnL, this interaction was further mapped to its 68-residue autonomously folding N-terminal segment, CdnLNt [10]. We therefore tested if CarD_1–72_, the CarDNt segment (residues 1 to 72) that aligns with CdnLNt, can on its own interact with RNAP-β. A striking difference between CarDNt and CdnL is that the former also interacts with CarG [5,8,11]. Hence, we also tested if this was mediated by CarD_1–72_ or by the C-terminal part of CarDNt spanning residues 61 to 179, CarD_61–179_ (Fig. 1A, 1B), which aligns with the corresponding CdnL segment, CdnLCt, shown to form a stable autonomously folded domain [10]. In BACTH analysis, CarD_1–72_ was found to mediate interaction only with RNAP-β, while CarD_61–179_ did so only with CarG (Fig. 2). Thus, two contiguous modules in CarDNt are implicated in two distinct protein-protein interactions: the N-terminal module CarD_1–72_ specifically targets RNAP and the remaining C-terminal part, CarD_61–179_, recognizes CarG.

### Structural analysis of CarDNt

**Fig 3:**
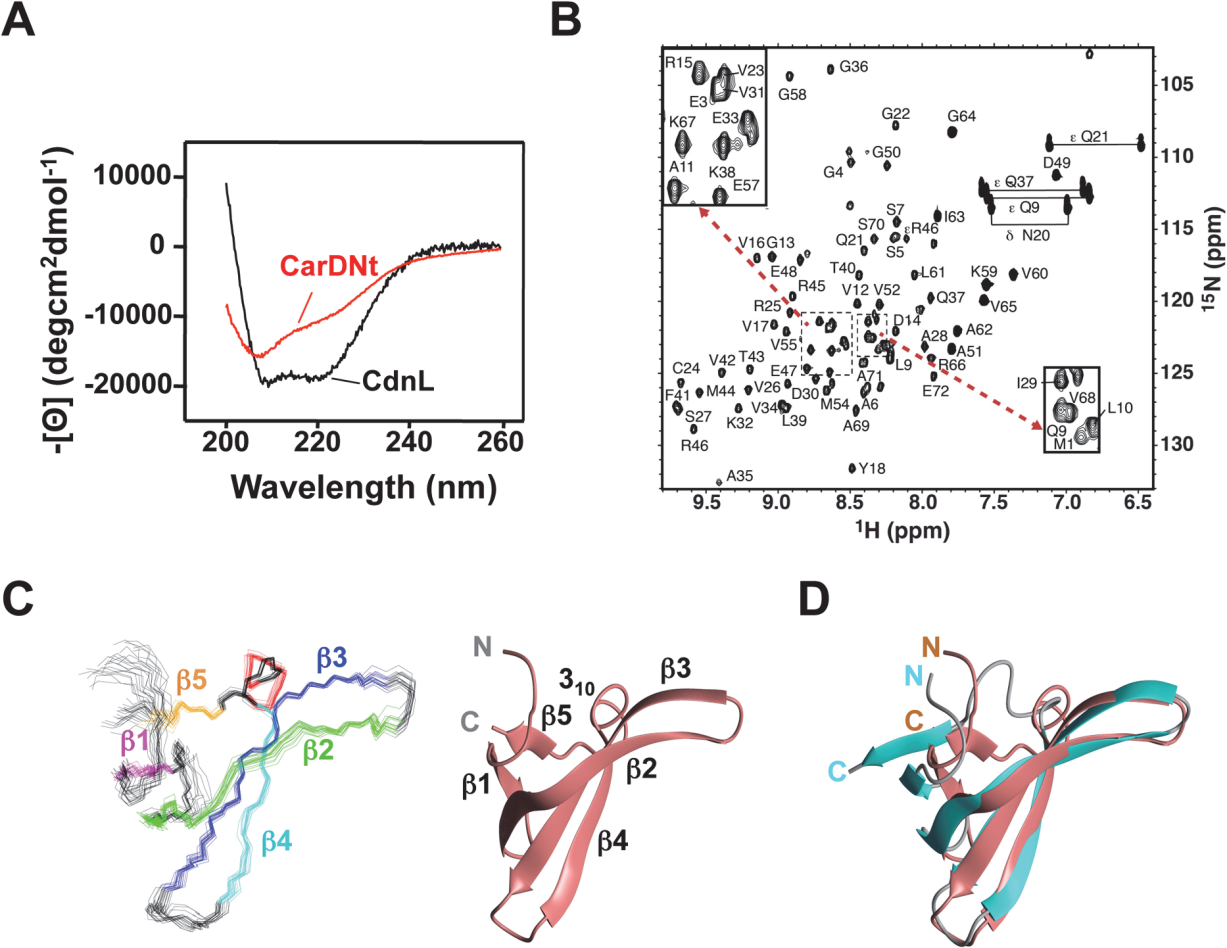
Structural analysis of CarDNt. (**A**) Far UV-CD spectra of CarDNt (red) and CdnL (black). (**B**) 2D [^1^H,^15^N]-HSQC spectrum of [^15^N,^13^C]-labeled CarD_1–72_. Crosspeak assignments are labeled with residues numbered as in the native protein, without including the non-native N-terminal AGH remaining after intramolecular cleavage of the intein-fusion tag. Crosspeaks for Q and N side chain amides are linked by a line and denoted δ and ε, respectively, and folded R NεH cross-peaks are shown as ε. Insets correspond to a zoom of the crowded region (boxes with dashed lines) as indicated. Unlabeled cross-peaks are those of a 15-residue peptide from the intein tag, generated upon its cleavage, which persisted despite extensive dialysis; these were detected due to their narrow line-widths and have chemical shifts close to random coil values (see [20]). (**C**) Left: superposition of the backbone traces for the 20 final NMR structures of CarD_1–72_ showing the β-strands β1 (magenta), β2 (green), β3 (blue), β4 (cyan) and β5 (gold) and a 3_10_ helix (red). Right: ribbon representation of the average CarD_1–72_ NMR solution structure with its β-strands and 3_10_-helix labeled. (**D**) Backbone overlay of the average NMR solution structure of native CarD_1–72_ (in coral) onto the average NMR structure of CdnLNt (cyan; PDB code: 2LT4). Structural representations were generated with MOLMOL.

Sequence-based secondary structure predictions suggest close correspondence between CarDNt and CdnL, with five β-strands and a short helix within the N-terminal stretch, and five helices in the C-terminal part [10]. These predictions were in consonance with the NMR solution structure determined for *M*. *xanthus* CdnL, where it was also shown that the β-strands segregate as a protease susceptible N-terminal domain and the α-helices as a protease-resistant C-terminal module [10]. Using far-UV CD spectroscopy, we experimentally compared the correspondence between CarDNt and CdnL in their secondary structures (Fig. 3A). This indicated a helix content for CarDNt of ~28% (calculated from the mean residue ellipticity at 222 nm, [Θ]_222_, of −9900 in degcm^2^dmol^−1^; see Materials and Methods), notably lower than the ~50% helix content ([Θ]_222_ = −18,200 degcm^2^dmol^−1^) estimated for CdnL. Hence, even though sequence-based analysis predicts similar secondary structures for CarDNt and CdnL, far-UV CD spectroscopy suggests differences between the two.

We next examined CarDNt structure at a higher resolution using NMR. Considerable signal broadening and overlapping peaks in the CarDNt NMR spectra complicated its analysis. A similar problem observed with CdnL was resolved by examining its isolated domains [10]. We therefore tested this approach with CarDNt. CarD_1–72_ could be stably expressed and its NMR spectra exhibited good overall quality and signal dispersion (Fig. 3B). Consequently, over 98% of its ^1^H, ^15^N, and ^13^C peaks could be assigned and its tertiary solution structure could be readily determined. This was not possible, however, with CarD_61–179_ due to its low stability, which is in agreement with earlier limited proteolysis data for CarD not detecting CarD_61–179_ as a stable domain [7]. By contrast, limited proteolysis of CdnL under similar experimental conditions indicated the segment equivalent to CarD_61–179_, CdnLCt, to be a stable domain [10], again consistent with structural differences between CarDNt and CdnL.

The CarD_1–72_ NMR structural ensemble was well-defined with the pair-wise root-mean-square deviations (rmsd) being (0.7± 0.2) Å for the structurally ordered backbone segments between residues 9 and 67 (Table B in S1 File). This region in CarD_1–72_ is composed of five antiparallel β-strands spanning residues 15–17, 23–34, 37–46, 51–56, and 65–67, respectively, in a twisted β-sandwich fold with a β5-β1-β2-β3-β4 topology and a 3_10_-helix between β4 and β5 (Fig. 3C). Most of the expected anti-parallel β-sheet cross-strand H-bonds were detected based on the criteria that the N-H…O donor-acceptor bond distances were ≤2.4 Å, and that the N-H…O bond angle deviations from 180° were <35°. The NMR structure of CarD_1–72_ closely matches that of CdnLNt determined previously [10], with maximum structural overlap in the β2-β3-β4 segment (rmsd of 2.5 Å for superposition of β1-β2-β3-β4-β5 and 1.2 Å for β2-β3-β4 backbone atoms; Fig. 3D). A database search for structural homologs of native CarD_1–72_ using DALI [35] yielded as the best matches (*Z-*scores >5) the equivalent modules in CdnL and its homologs [18,20–22] and *E*. *coli* TRCF-RID, and to a lower degree (*Z-*scores <4.5), various other proteins with Tudor-like domains [36,37]. The close structural similarity of CarD_1–72_ and CdnLNt suggests that the different secondary structures for CarDNt and CdnL based on far-UV CD likely stem from differences between CarD_61–179_ and CdnLCt.

### Mutations disrupting CarD interactions with RNAP and its consequences *in vivo*

**Fig 4:**
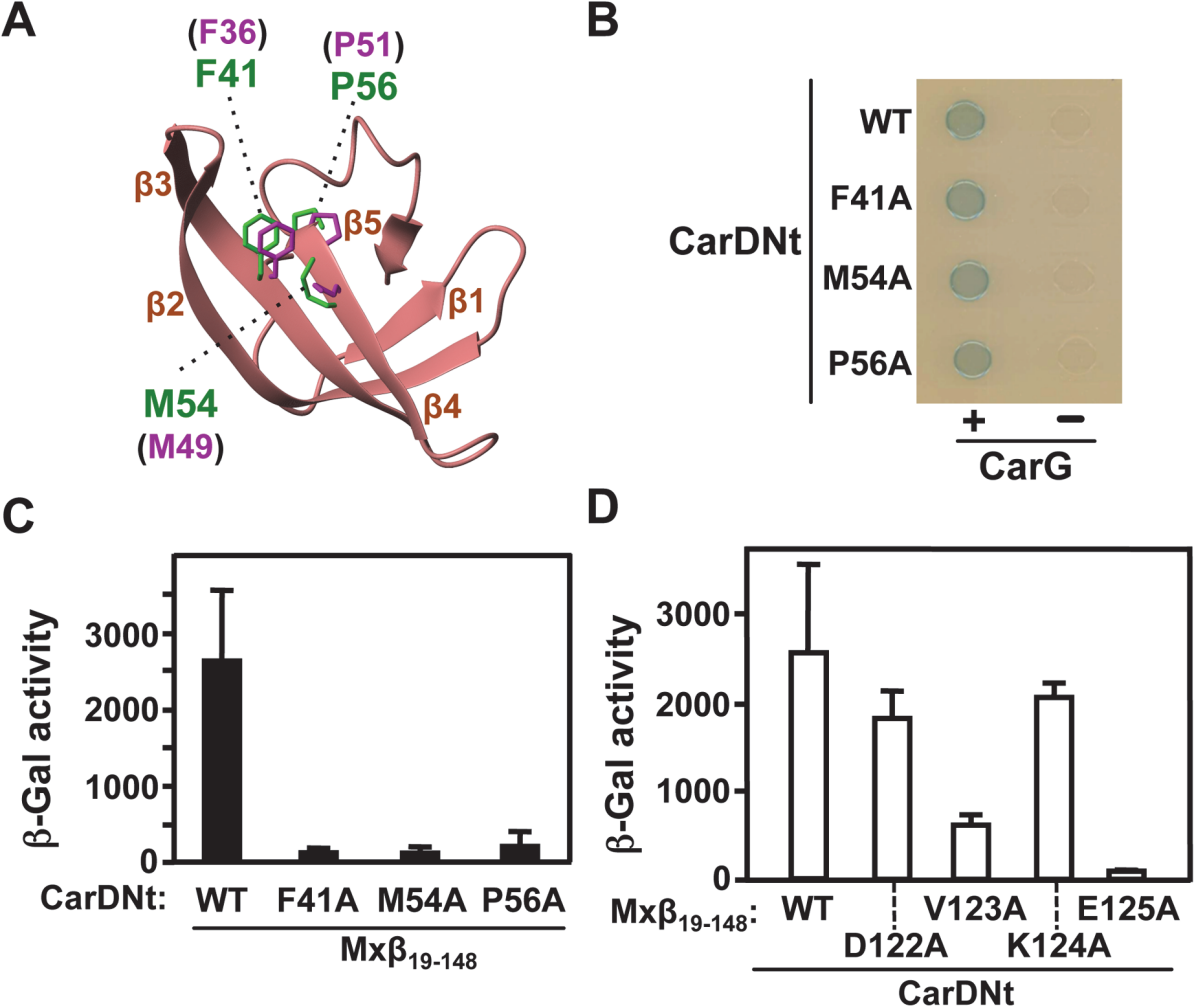
Mutational analysis of CarDNt interactions with RNAP-β. (**A**) Ribbon representation of the average CarD_1–72_ NMR solution structure showing residue side chains (labeled in green) tested by site-directed mutagenesis, whose equivalents in CdnL (labeled in magenta) contact RNAP-β (see text). The sidechains are represented based on the CarD_1–72_ and CdnL backbones corresponding to an overlay of their β2-β3-β4 segments. The structure was generated using MOLMOL. (**B**) BACTH analysis (spot assay) of the interaction between the indicated CarDNt mutants expressed in pKT25 and CarG in pUT18C. (**C**) BACTH analysis of the interaction between the indicated CarDNt mutants expressed in pKT25 and Mxβ_19–148_ in pUT18C. (**D**) BACTH analysis of the interaction between the indicated Mxβ_19–148_ mutants expressed in pUT18C versus CarDNt in pKT25. In **C** and **D** the mean and standard error of three independent experiments are shown.

Given their similar tertiary structures and shared ability to interact with RNAP-β, we tested if CarD_1–72_ also mirrors CdnLNt in specific contacts with RNAP-β. In CdnL, mutating F36, M49, or P51 to alanine did not affect its protein stability or folding but abrogated interaction with RNAP-β [10]. The corresponding CarD residues are F41, M54 and P56 (Fig. 1A, Fig. 4A). No effect on the interaction with CarG, as verified by BACTH analysis (Fig. 4B), nor on CarD stability (see below), was observed when any of the three residues was mutated to alanine, suggesting that these mutants were stably expressed and properly folded. However, all three CarDNt mutants were impaired in the interaction with Mxβ_19–148_ (Fig. 4C), indicating that CdnL contacts with RNAP-β appear to be conserved in CarD.

In the *M*. *xanthus* RNAP-β segment D122-V123-K124-E125, which is quite conserved in various RNAP-β, mutating (to alanine) V123 or E125, but not D122 or K124, impaired the interaction with CdnL [10]. Hence, we tested if this segment was also important for the interaction with CarDNt. BACTH analysis indicated that, just as with CdnL, the interaction of Mxβ_19–148_ with CarDNt was impaired on mutating V123 or E125, but not D122 or K124 (Fig. 4C). Thus, *M*. *xanthus* RNAP-β appears to employ equivalent contacts to recognize CarDNt and CdnL. Interestingly, despite the finding here that CarDNt mimics CdnL in its mode of interaction with RNAP, only the latter stabilizes RP_o_ formation at an rRNA promoter *in vitro* [10].

**Fig 5:**
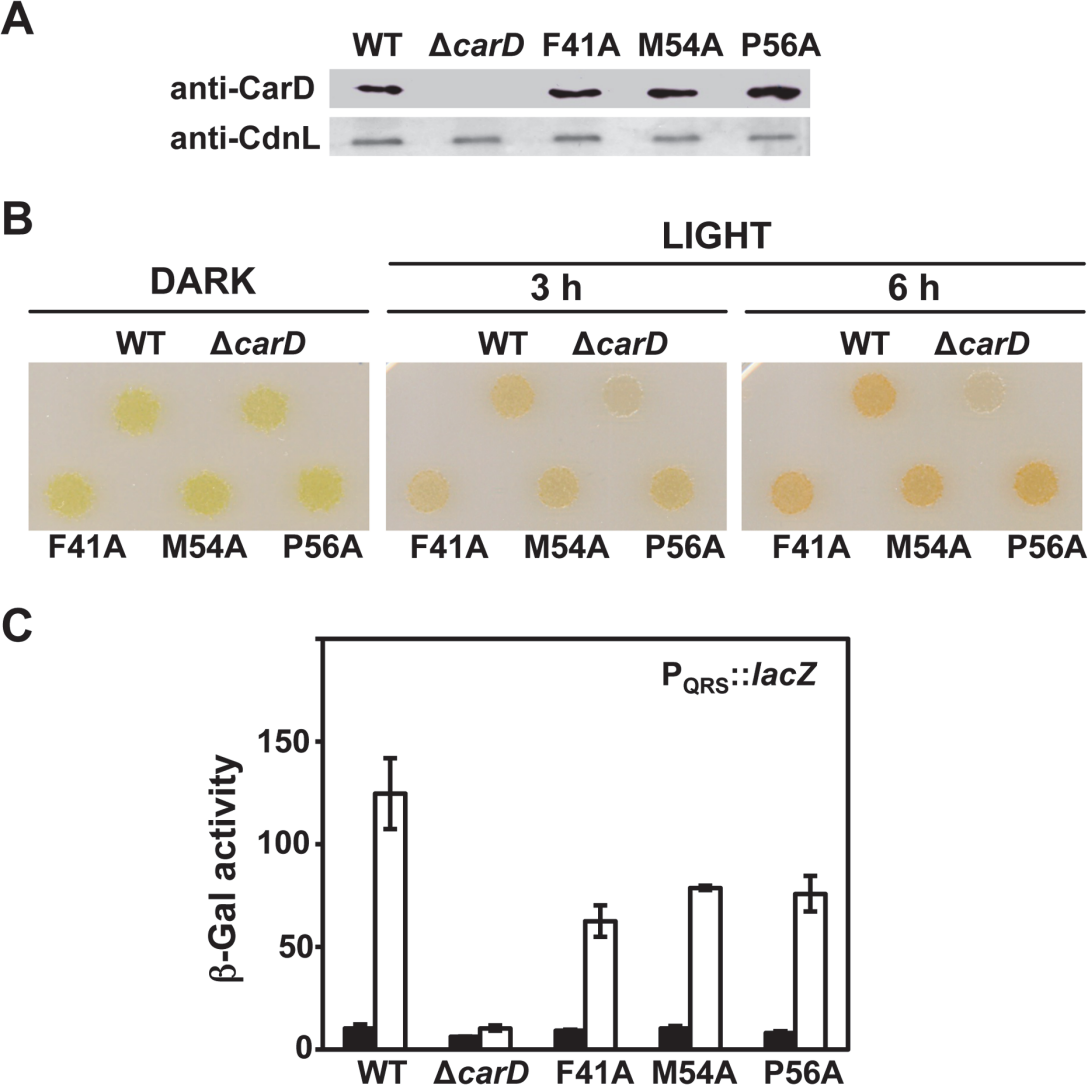
Consequences *in vivo* of disrupting CarD interactions with RNAP. (**A**) Western blots of cell extracts of *M*. *xanthus* strains expressing wild-type (WT) *carD* (strain MR1901), Δ*carD*, or the indicated mutant *carD* allele using monoclonal anti-CarD antibodies (top strip) and, as loading control, using polyclonal anti-CdnL antibodies (bottom strip). (**B**) Colony color phenotype of *M*. *xanthus* strains expressing wild-type (WT), Δ*carD*, or the indicated mutant *carD* allele. Here, 5 μl drops from each liquid culture (OD_550_ ~0.8) were spotted on two CTT plates, grown for 12 hr at 33°C in the dark, after which one plate continued to be incubated in the dark while the other was exposed to light. (**C**) Reporter P_QRS_::*lacZ* expression (β-Gal activity) measurements for exponentially growing cells of the wild-type (WT) and the indicated *carD* mutant strains in the dark (filled bars) or after 8 hours in the light (unfilled bars). The mean and standard error of three independent experiments are shown.

Mutations in CdnL that disrupt the interaction with RNAP have been found to seriously impair its function in *M*. *xanthus*, affecting cell growth and survival [10]. We therefore tested the consequences of such mutations in CarD, focussing on its effect on light-induced carotenogenesis and the CarD-dependent activation of the P_QRS_ promoter. For this, *M*. *xanthus* strains with endogenous *carD* substituted by the F41A, M54A or P56A mutant *carD* alleles were generated (see Materials and Methods). Western blots of the corresponding cell extracts using monoclonal anti-CarD antibodies confirmed stable expression of all three mutant proteins in *M*. *xanthus* (Fig. 5A). Surprisingly, the F41A, M54A or P56A mutant strains remained Car^+^ despite their impaired interactions with RNAP (Fig. 5B). The light-induced expression levels of the reporter P_QRS_-*lacZ* probe in all three mutant strains were comparable and about 60–70% of that in wild-type cells (Fig. 5C). Since CarDNt alone exhibited partial activity, the effects of the F41A and M54A mutations were also tested with only this domain present *in vivo*. We found that the response to light of the strain expressing CarDNt persisted when its interaction with RNAP was impaired by the above mutations (Fig. B in S1 File). This was observed even with the double F41A/M54A mutant, for which the reporter P_QRS_-*lacZ* activity estimated was comparable to that for the wild-type CarDNt (Fig. B in S1 File). Thus, loss of interaction with RNAP, which abrogates CdnL function, does not significantly affect CarD activity.

### **CarD**_61–179_ **plays at least two distinct and crucial roles in CarD function**

Elimination of CarDNt results in the same phenotype as that caused by the complete deletion. That disrupting its interaction with RNAP, which occurs via CarD_1–72_, did not abolish CarD activity emphasizes the critical role of CarD_61–179_ which, as shown above, mediates interaction with CarG. Interestingly, the equivalent region of CdnL (CdnLCt), which does not interact with CarG, is also indispensable [10]. Several residues in a solvent-exposed basic-hydrophobic region of the overall acidic CdnLCt have been mutated to alanine in CdnL: the nonpolar W88 (highly conserved), M96 and F125 (most solvent-exposed), as well as the basic residues R128/K129 and R90/R91/R93 (see Fig. 1A). The conserved W and the basic patch have been proposed to mediate the non-specific DNA-binding exhibited by the mycobacterial CdnL and their mutations shown to impair function [18,19,21]. By contrast, CdnL (or CdnLCt) did not exhibit DNA binding activity *in vitro* and mutation of the highly conserved W or of the basic R128/K129 residues did not have a drastic effect; only the F125A and the R90A/R91A/R93A mutations produced noticeable effects, the triple mutant being almost equivalent to lack of CdnL *in vitro* and *in vivo* [10].

**Fig 6:**
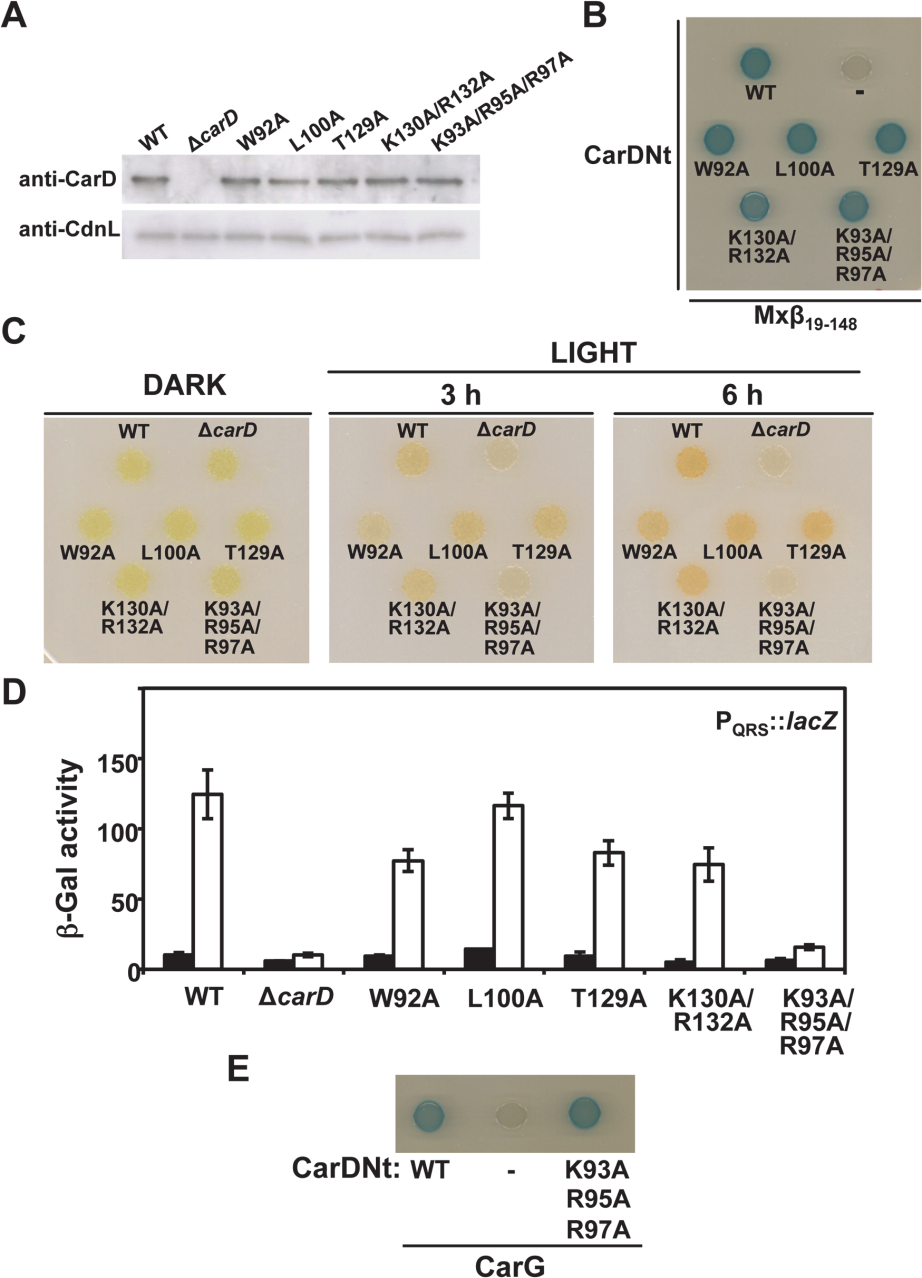
Consequences of site-directed mutations in the CarD_61__–__179_ segment of CarD on its function *in vivo*. (**A**) Western blots of cell extracts of *M*. *xanthus* strains expressing wild-type (WT) *carD* (strain MR1901), Δ*carD*, or the indicated mutant *carD* allele using monoclonal anti-CarD antibodies (top strip) and, as loading control, using polyclonal anti-CdnL antibodies (bottom strip). *(* **B**) BACTH analysis of the interaction between the indicated CarDNt versions expressed in pKT25 and Mxβ_19–148_ in pUT18C. “-” is the negative control, with pKT25-CarDNt and empty pUT18C vector. *(* **C**) Colony color phenotype of *M*. *xanthus* wild-type (WT), Δ*carD*, and the indicated *carD* mutant strains analyzed as in Fig. 5B. *(* **D**) Reporter P_QRS_::*lacZ* expression (β-Gal activity) measurements for exponentially growing cells of each of the indicated strains in the dark (filled bars) or after 8 hours under light (unfilled bars). The mean and standard error of three independent experiments are shown. *(* **E**) BACTH analysis confirming interaction of the CarD K93A/R95A/R97A mutant (in pKT25) with CarG (in pUT18).

For comparison with equivalent mutations in CdnL, we mutated the following residues in CarD: W92, L100 and T129 (corresponding, respectively, to the solvent-exposed, nonpolar W88, M96, and F125 in CdnL), K130/R132 (R128/K129 in CdnL), and K93/R95/R97 in CarD (corresponding to R90/R91/R93 in CdnL) (Fig. 1A). The CarD mutant proteins were detected in Western blots of cell extracts of *M*. *xanthus* strains in which the wild-type gene was replaced by each of these mutant *carD* alleles (Fig. 6A) and they interacted with Mxβ_19–148_ in BACTH analysis, as expected (Fig. 6B). Hence, these CarD variants are stably expressed and folded. When analyzed for light-induced carotenogenesis in *M*. *xanthus*, only the K93A/R95A/R97A triple mutant was Car^-^ (Fig. 6C). Moreover, light-inducible reporter P_QRS_-*lacZ* activity was observed for all the mutants at 60–90% of wild-type levels, except for the K93A/R95A/R97A mutant, whose *lacZ* activity was as low as that of the Δ*carD* strain (Fig. 6D). BACTH analysis indicated that the interaction with CarG remains intact for the K93A/R95A/R97A mutant (Fig. 6E), implying that these basic residues play a critical role in CarD function that is distinct from the interaction with CarG. When the effect of the K93A/R95A/R97A mutation was assessed in the context of the partial activity of CarDNt *in vivo*, the triple mutation again caused a loss of the ability to produce carotenoids in the light with negligible light-inducible reporter P_QRS_-*lacZ* activity (Fig. B in S1 File), in contrast to the strains with wild-type CarDNt or its K130A/R132A variant. The strong negative effect of the triple mutation on CarD activity mirrors that of equivalent mutations in CdnL (which does not interact with CarG) suggesting that these basic residues may play analogous functional roles in CarD and CdnL.

Since R95 is the residue that is most highly conserved within the K93/R95/R97 stretch in all CarD and CdnL homologs, we also checked the effect of mutating only this arginine to alanine on CarD function. We found that light-induced carotenogenesis and reporter P_QRS_-*lacZ* activity was considerably diminished in the R95A mutant, albeit somewhat less than with the K93A/R95A/R97A mutant (Fig. C in S1 File). This suggests that R95 is important but K93 and/or R97 also likely contribute to the crucial role of this segment in CarD function.

In sum, our results indicate that the CarD_61–179_ part of CarDNt mediates at least two functionally critical activities in the expression of *carQRS*: the specific interaction with CarG, and that mediated by the K93/R95/R97 segment, whose exact role remains to be defined. The equivalent domain of CdnL is also indispensable for its distinct function, which is required for cell growth, and this CdnL domain conserves the functionally crucial basic residue segment but not the interaction with CarG.

## Discussion

The *M*. *xanthus* regulatory protein CarD and CdnL are prototypical members of the widespread CarD_CdnL_TRCF family of bacterial RNAP-binding proteins that have been implicated, respectively, in the action of several ECF-σ factors and in σ^A^-dependent rRNA promoter activation [2,4,9–11]. The molecular bases for their distinct functions, however, remain elusive. A systematic dissection of their structures and interactions can provide insights into their distinct modes of action, and the present study describes such an analysis with CarDNt, the ~180-residue CarD N-terminal region that is similar to CdnL in sequence. CarDNt is the structurally defined part of CarD that does not bind to DNA, in contrast to the remaining intrinsically unfolded C-terminal HMGA-like region (absent in CdnL) that preferentially binds to the minor groove of AT-rich DNA tracts [7]. Unlike the HMGA-like domain, which alone is completely inactive *in vivo*, we find that CarDNt has observable activity on its own, albeit at lower levels than full-length CarD. CarDNt is thus sufficient for CarD function, while the HMGA domain is required to maximize activity.

Our data reveal two protein-protein interaction regions in CarDNt, each directed at a distinct protein partner. One mediates binding to RNAP-β and corresponds to the 72-residue N-terminal segment, CarD_1–72_, whose twisted, five-stranded β-sheet Tudor-like fold as well as contacts with RNAP-β closely match those observed for its CdnL counterpart. The second CarDNt module, CarD_61–179_, interacts with CarG, but this protease-susceptible domain has thus far eluded a high-resolution structure determination. By contrast, the CarD_61–179_ counterpart of CdnL, CdnLCt, is protease-resistant, could be expressed as a stable isolated domain, and its compact, all-helical, high-resolution tertiary structure readily determined by NMR [10]. These differences and the inferences from far-UV CD data suggest that CarD_61–179_ and CdnLCt are likely to be structurally distinct. Even so, CarD_61–179_ has a stretch of basic residues that is not involved in the interaction with RNAP or CarG and yet is critical for CarD function, just as, interestingly, the equivalent conserved segment in CdnLCt is crucial for CdnL function. In sum, our data indicate that CarDNt mirrors CdnL in domain organization and in various interactions, but different contributions from these and additional interactions specific to CarD can account for its distinct function.

Mutations that impair CdnL interactions with RNAP-β were very adverse to cell growth and survival [10]. It is therefore interesting that equivalent mutations in CarD, which mimics CdnL in RNAP-β recognition, only slightly lowered its activity *in vivo*. Studies with CdnL showed that it associates with rRNA promoters *in vivo* and activates these by stabilizing the formation of transcriptionally competent open complexes (RP_O_) by RNAP holenzyme with the primary housekeeping σ^A^; and that this activity of CdnL is impaired by mutations disrupting the interaction with RNAP-β [10,12,18,19]. CdnL did not preferentially localize *in vivo* at P_QRS_, the alternative ECF-σ CarQ-dependent promoter whose activation requires CarD, suggesting absence of direct CdnL action at this promoter [10]. By contrast, both CarD and CarDNt act on P_QRS_ but not on rRNA promoters [4,10]. The little or no effect on P_QRS_ expression on disrupting CarD/CarDNt binding to RNAP-β therefore suggests that this interaction, unlike with CdnL, is not as critical in transcriptional activation mediated by CarD. Other CarD interactions should then be more decisive determinants of its function.

One interaction indispensable in every known CarD-dependent process is that with CarG [4,5,8]. We mapped this interaction in the present study to the CarD_61–179_ segment, but the exact molecular mechanism by which CarG acts together with CarD in ECF-σ promoter activation remains enigmatic. In the HMGA-driven assembly of the large transcriptionally competent complex in eukaryotes known as the enhanceosome, a key role is played by transcriptional factors that do not bind DNA but rather provide a protein scaffold for interaction with various other regulatory factors [38,39]. CarG, which does not bind DNA directly, could collaborate with CarD in mediating the recruitment of additional factors required for promoter expression or bridge additional contacts with the basal transcriptional machinery essential for activation. We have not been able to detect direct CarG physical interactions with any of the core RNAP subunits nor with CarQ in two-hybrid analysis [29], but the likelihood that these occur once the CarD/CarG complex and RNAP have assembled at the target promoters cannot be discarded. If so, such interactions involving CarG might explain why disrupting that of CarD with RNAP-β had no dramatic effect on function.

A second characteristic CarD interaction is that mediated by its HMGA-like C-terminal domain. This domain confers an intrinsic DNA binding capability to CarD *in vitro* that we have thus far never detected with either CarDNt or CdnL [10]. Hence, the HMGA-like domain probably mediates the *in vivo* nucleoid localization of CarD [8], whereas that of CdnL might occur via piggyback recruitment by RNAP [11]. The colocalization *in vivo* of mycobacterial CdnL and RNAP σ^A^ and its absence from non-promoter regions also suggested that CdnL is targeted to the genome through its interaction with RNAP and not through direct binding to DNA [40]. The CarD HMGA-like domain resembles its eukaryotic counterpart in binding preferentially to the minor groove of AT-rich tracts of appropriate length and spacing, and can be replaced by other basic and, necessarily, intrinsically disordered domains with no loss of function *in vivo* [5,7,29]. The contribution of this domain and the DNA-binding ability it confers in CarD function is further demonstrated by the reduced CarD activity *in vivo* on mutating an AT-rich site lying upstream of the ECF-σ promoter P_QRS_ to which CarD exhibits minor groove binding via its HMGA-like domain [6,7,29,41] or, as shown in this study, on deleting the CarD HMGA-like DNA-binding domain. Interactions of eukaryotic HMGA with DNA, various factors and the basal machinery have been shown to synergistically favor the cooperative assembly of the enhanceosome, and it has been proposed that HMGA functions as a DNA chaperone to stabilize a DNA structure resembling that in the fully assembled enhanceosome [38,39,42]. Thus, it is conceivable that, in an analogous manner, DNA binding via the HMGA-like domain also synergistically enhances the low level activity of CarDNt by favouring a particular DNA conformation at the target promoter.

It is remarkable that, despite the divergences discussed above between CarDNt and CdnL, both have a conserved basic residue segment crucial for their respective functions. With CdnL, its ability to stabilize RP_O_ formation at rRNA promoters was abolished on mutating the basic segment [10]. The details on what exactly the basic segment does in the RP_O_ complex and whether it has a similar role in CarD function, however, remain open. This basic segment and an adjacent highly conserved tryptophan have been linked to DNA binding based on the weak, nonspecific binding to double-stranded DNA observed *in vitro* with the mycobacterial CdnL homolog, and single mutations of the basic residues or the tryptophan were detrimental for *M*. *tuberculosis* growth and viability [18,19,21]. In *M*. *xanthus*, mutation of the basic segment, but not of the tryptophan, drastically impaired CarD function *in vivo*, just as was observed with CdnL. Thus, while the conserved tryptophan appears to play an important but unknown role in the function of the *M*. *tuberculosis* protein, it does not appear to do so in *M*. *xanthus* CarD or CdnL. On the other hand, the basic residues are functionally crucial in both *M*. *xanthus* proteins as well as in that of *M*. *tuberculosis*, even though in contrast to the latter neither CarDNt nor *M*. *xanthus* CdnL exhibited any intrinsic DNA binding *in vitro*. That CdnL with its intact basic segment is required in RP_O_ formation hints that any crucial and specific contacts potentially involving this segment would more likely be with elements within RP_O_ (not naked DNA), and structural models have been proposed for possible interaction in RP_O_ with the transcription bubble or RNAP-σ^A^ [18,22]. It is tempting to speculate that CarD and its crucial basic segment play a role at its specific ECF-σ target promoter complexes analogous to that of CdnL at σ^A^ promoters, but resolving these issues for CarD or CdnL would require further higher resolution analyses in future studies. Nevertheless, the structure-function dissection of CarDNt reported here has highlighted four distinct interactions associated with CarD and their relative functional importance: those involving CarG and a conserved basic residue segment in CarD_61–179_ are crucial for CarD function, that with RNAP is apparently dispensable, and that with DNA via the HMGA-like domain contributes synergistically to maximize activity.

In an early study, we speculated that CarD may have evolved from an interkingdom domain fusion of a preexisting bacterial domain akin to CarDNt or CdnL and an eukaryotic HMGA-like domain acquired by horizontal gene transfer [9]. Subsequent analysis of the rather large (among bacteria) *M*. *xanthus* genome suggested that gene duplication, divergence, horizontal gene transfer and domain fusion events may have occurred quite extensively in the evolution of the sensory complexity of this bacterium [43]. The similar bimodular organization and interactions in CarDNt and CdnL, the matching structures and interaction with RNAP of their N-terminal modules, and a conserved and functionally crucial segment in the C-terminal module are all consistent with the proposal that CarD and CdnL evolved from a common ancestral protein. Also, both proteins are implicated in promoter activation dependent on related yet distinct σ factors. Acquisition of the additional HMGA-like domain and the interaction with CarG by CarDNt, which retains various features of CdnL, must have then enabled the functional diversification characterizing CarD. Tellingly, available genome data suggest that CarG as well as the HMGA domain exist only in CarD-containing myxobacteria, whereas CdnL homologs occur not only in all myxobacteria but also in numerous other bacterial species. Our modular dissection of CarDNt structure and interactions, their contributions to CarD function, and comparisons with CdnL therefore highlight the common structural modules and interactions shared by the two proteins, as well as the additional interactions and domains that have evolved in CarD to enable the distinct functions of these two related members of a large and important bacterial protein family.

## Accession Numbers

Accession codes for CarD_1–72_ are 2LT1 for structural coordinates deposited in the Protein Data Bank, and 18194 for NMR chemical shifts in BioMagResBank (http://www.bmrb.wisc.edu/).

## Supporting Information

S1 File Contains Table A and Table B, Fig. A, Fig. B, and Fig. C with legends, and References. (DOC)
