# Supplementary material for: Structure-Function Dissection of Myxococcus xanthus CarD N-Terminal Domain, a Defining Member of the CarD_CdnL_TRCF Family of RNA Polymerase Interacting Proteins
Source: PLoS One. 2015 Mar 26;10(3):e0121322. doi: 10.1371/journal.pone.0121322 (PMC4374960; doi:10.1371/journal.pone.0121322)
Supplement: S1 File — (DOC) [file pone.0121322.s001.doc]

**SUPPORTING INFORMATION**

**Structure-function dissection of *Myxococcus xanthus* CarD N-terminal domain, a defining member of the CarD_CdnL_TRCF family of RNA polymerase interacting proteins**

Diego Bernal-Bernal^1^, Aránzazu Gallego-García^1^, Gema García-Martínez^1^, Francisco García-Heras^1^, María Angeles Jiménez^2^*, S. Padmanabhan^2^*, Montserrat Elías-Arnanz^1^*

^1^ Departamento de Genética y Microbiología, Área de Genética (Unidad Asociada al IQFR-CSIC), Facultad de Biología, Universidad de Murcia, 30100 Murcia, Spain

^2^ Instituto de Química Física ‘Rocasolano’, Consejo Superior de Investigaciones Científicas, Serrano 119, 28006 Madrid, Spain

*Address correspondence to Montserrat Elías Arnanz (E-mail: melias@um.es), María

Angeles Jiménez (E-mail: majimenez@iqfr.csic.es), or to S. Padmanabhan (E-mail:

padhu@iqfr.csic.es).

**Table A. *Myxococcus xanthus* strains and plasmids used in this work**

| ***M. xanthus* strain** | **Description** | **Source / Reference** |
| --- | --- | --- |
| DK1050 | Wild-type *M. xanthus* strain |  |
| DK1622 | Wild-type *M. xanthus* strain |  |
| MR1316 | ∆*ddvA* |  |
| MR1317 | ∆*ddvA ∆carD* |  |
| MR1900 | *carD3* (Δ*carD*) |  |
| MR1901 | *carD´* (*carD* reinserted at the endogenous site) |  |
| MR1908 | Δ*carDNt* (*carD_180-316_* inserted at the endogenous site; merodiploid) |  |
| MR2222 | *carDNt* | This work |
| MR2226 | *carDNt(F41A)* | This work |
| MR2227 | *carDNt(M54A)* | This work |
| MR2228 | *carDNt(F41A/M54A)* | This work |
| MR2229 | *carDNt(W92A)* | This work |
| MR2230 | *carDNt(K93A/R95A/R97)* | This work |
| MR2231 | *carDNt(T129A)* | This work |
| MR2232 | *carDNt(K130A/R132A)* | This work |
| MR2240 | Δ*carDNt* (haploid derived from MR1908) | This work |
| MR2257 | *carD(F41A)* | This work |
| MR2258 | *carD(M54A)* | This work |
| MR2259 | *carD(P56A)* | This work |
| MR2280 | *carD(W92A)* | This work |
| MR2281 | *carD(L100A)* | This work |
| MR2282 | *carD(T129A*) | This work |
| MR2283 | *carD(K130A/R132A)* | This work |
| MR2284 | *carD(K93A/R95A/R97A)* | This work |
| MR2755 | *carD(R95A)* | This work |
| MR2756 | ∆*ddvA carDNt* | This work |
| MR2759 | ∆*ddvA* ∆*carDNt* | This work |
| **Plasmid** | **Description** | **Source / Reference** |
| pDAH217 | Vector to introduce the P_QRS_::*lacZ* reporter probe into *M. xanthus*. Km^R^ |  |
| pKT25 | Vector for C-terminal fusion constructs to the T25 fragment of CyaA for use in bacterial two-hybrid analysis. Km^R^ |  |
| pTYB12 | Vector for overexpressing proteins with an N-terminal intein tag. Amp^R^ | NE Biolabs |
| pUT18 | Vector for N-terminal fusion constructs to the T18 fragment of CyaA for bacterial two-hybrid analysis. Amp^R^ |  |
| pUT18C | Vector for C-terminal fusion constructs to the T18 fragment of CyaA for bacterial two-hybrid analysis. Amp^R^ |  |
| pMR2603 | In-frame *carD* deletion (Δ*carD*). To insert *carD* variants for complementation analysis. Km^R^Gal^S^ |  |
| pMR2696 | pMR2603-*carD* |  |
| pMR2768 | Vector to insert *carD_180-316_* (Δ*carDNt*) at the endogenous *carD* locus |  |
| pMR2880 | pUT18-*carG* |  |
| pMR2881 | pKT25 with gene for CarDNt |  |
| pMR2973 | pKT25-*cdnL* |  |
| pMR3034 | Vector to introduce the P*_ddvS_*::*lacZ* reporter probe into *M. xanthus*. Km^R^ |  |
| pMR3389 | pMR2603-*carDNt* | This work |
| pMR3397 | pTYB12 construct to overexpress CarDNt | This work |
| **Plasmid** | **Description** | **Source / Reference** |
| pMR3412 | pUT18C with gene for Mxβ_19-148_ |  |
| pMR3486 | pKT25 with gene for CarD_61-179_ | This work |
| pMR3585 | pUT18C with gene for β_19-148_(D122A) | This work |
| pMR3586 | pUT18C with gene for β_19-148_(V123A) | This work |
| pMR3587 | pUT18C with gene for β_19-148_(K124A) | This work |
| pMR3588 | pUT18C with gene for β_19-148_(E125A) | This work |
| pMR3633 | pTYB12 construct to overexpress CarD_1-72_ | This work |
| pMR3687 | pMR2603-*carD(F41A)* | This work |
| pMR3688 | pMR2603-*carD(M54A)* | This work |
| pMR3689 | pMR2603-*carD(P56A)* | This work |
| pMR3734 | pKT25 with gene for CarDNt(F41A) | This work |
| pMR3735 | pKT25 with gene for CarDNt(M54A) | This work |
| pMR3736 | pKT25 with gene for CarDNt(P56A) | This work |
| pMR4105 | pKT25 with gene for CarDNt(W92A) | This work |
| pMR4106 | pKT25 with gene for CarDNt(L100A) | This work |
| pMR4113 | pKT25 with gene for CarDNt(T129A) | This work |
| pMR4114 | pMR2603-*carD(W92A)* | This work |
| pMR4115 | pMR2603-*carD(L100A)* | This work |
| pMR4116 | pMR2603-*carD(T129A)* | This work |
| pMR4157 | pMR2603-*carD(K130A/R132A)* | This work |
| pMR4158 | pMR2603-*carD(K93A/R95A/R97A)* | This work |
| pMR4173 | pKT25 with gene for CarDNt(K130A/R132A) | This work |
| pMR4174 | pKT25 with gene for CarDNt(K93A/R95A/R97A) | This work |
| pMR4191 | pMR2603-*carDNt(F41A)* | This work |
| pMR4192 | pMR2603-*carDNt(W92A)* | This work |
| pMR4193 | pMR2603-*carDNt(T129A)* | This work |
| pMR4194 | pMR2603-*carDNt(K130A/R132A)* | This work |
| pMR4200 | pMR2603-*carDNt(M54A)* | This work |
| pMR4201 | pMR2603-*carDNt(F41A/M54A)* | This work |
| pMR4216 | pMR2603-*carDNt(K93A/R95A/R97A)* | This work |
| pMR4253 | pMR2603-*carD(R95A)* | This work |

Km^R^, kanamycin resistance; Gal^S^, galactose sensitivity due to galK; Amp^R^, ampicillin resistance.

**Table B. NMR structural statistics for the ensemble of the 20 lowest energy CarD_1-72_ structures^a^**

| **NOE distance constraints** | | |
| --- | --- | --- |
| Intraresidue (*i–j*=0) | 223 | |
| Sequential (|*i–j*|=1) | 173 | |
| Medium range (1<|*i–j*|<5) | 84 | |
| Long-range (|*i–j*| ≥5) | 219 | |
| Total number | 699 | |
| Averaged total per residue | 9.4 | |
| **Dihedral angle constraints** | | |
| φ angles | 67 | |
| ψ angles | 54 | |
| Total | 121 | |
| **Maximum constraints violations** | | |
| Distance (Å) | 0.17±0.02 | |
| Dihedral angle (º) | 3.5±0.8 | |
| **Averaged structure energies** | | |
| CYANA target function value | 0.45 ± 0.03 | |
| AMBER energy (kcal/mol) | − 2745 | |
| Van der Waals energy (kcal/mol) | − 445 | |
| Electrostatic energy (kcal/mol) | − 4564 | |
| **Deviations from ideal geometry** | | |
| Bond length (Å) | 0.013 | |
| Bond angle (º) | 1.9 | |
| **Pairwise rmsd (Å) (Backbone/Heavy atoms)** | | |
| All residues | | 1.3±0.3/1.8±0.2 |
| Ordered residues | | 0.7±0.2/1.3±0.2 |
| **Ramachandran analysis (ordered/all residues)** | | |
| Most favoured regions (%) | 87.1/82.2 | |
| Additional allowed regions (%) | 12.4/16.3 | |
| Generously allowed regions (%) | 0.6/1.5 | |
| Disallowed regions (%) | 0.0/0.0 | |

^a^PDB accession code 2LT1. Ordered residues (numbered from the N-terminus as in native CarD): 9 to 67, the N-terminal AGH cloning tag excluded from these analyses.

**
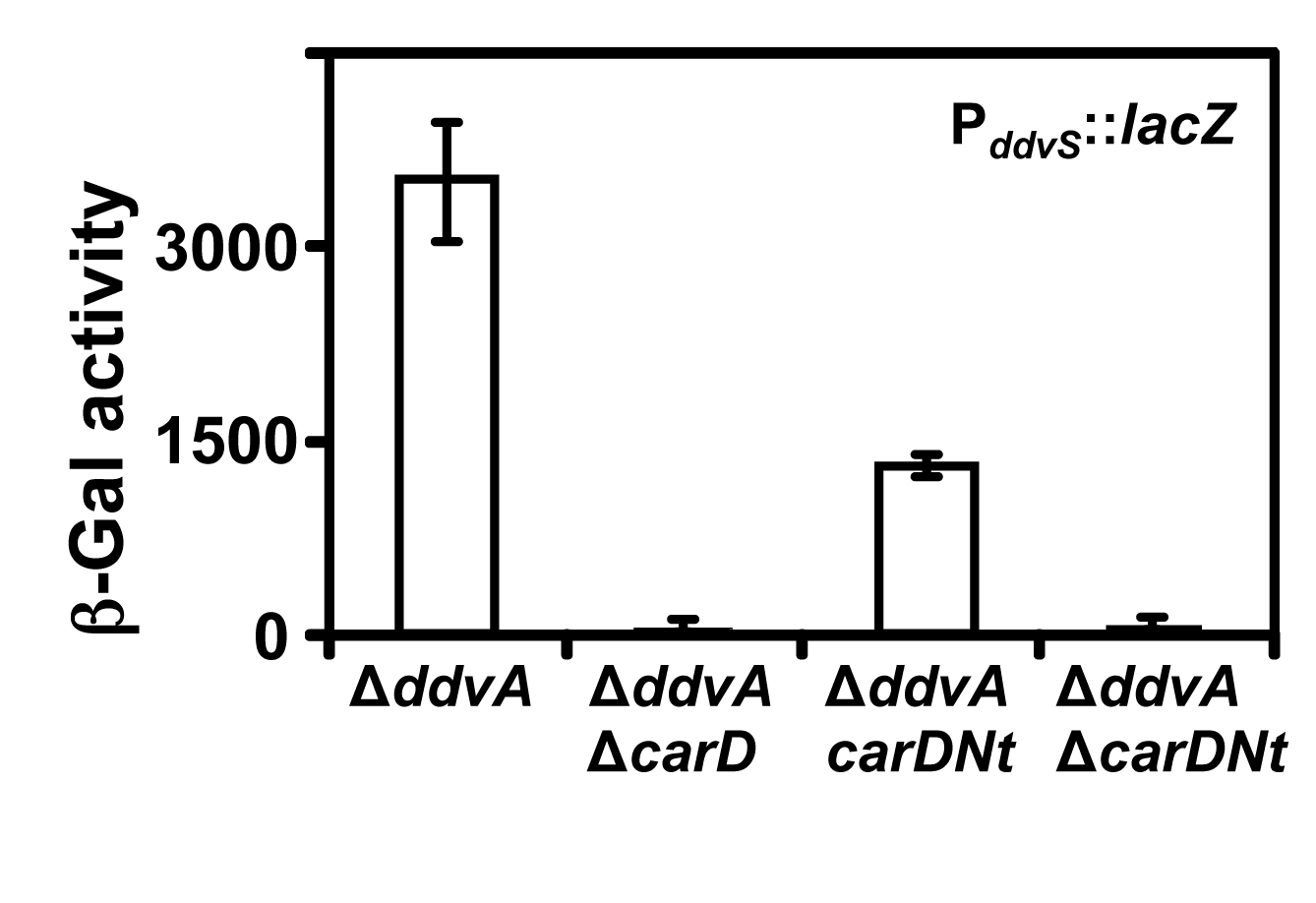
**

**Fig A.** **Effect of replacing CarD by CarDNt on CarD-dependent P*_ddvS_* activation *in vivo*.** Reporter P*_ddvS_*::*lacZ* expression (β-Gal activity) measurements for exponentially growing cells in the indicated strains. Because the natural signal that inactivates DdvA is still unknown, this analysis was carried out in a Δ*ddvA* genetic background, where lack of the anti-σ DdvA activates the ECF-σ DdvS.


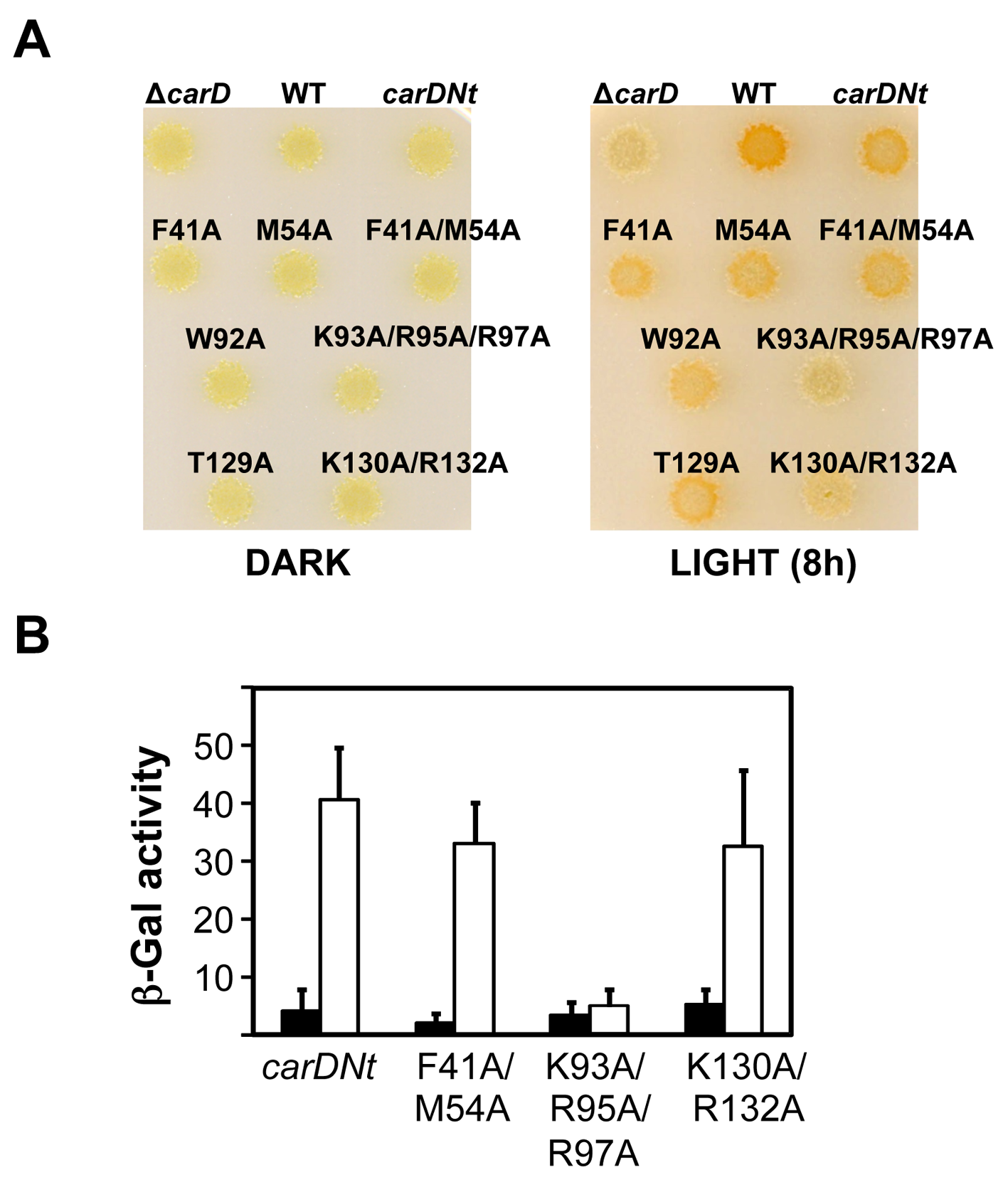


**Figure B. Light-induced carotenogenesis in *M. xanthus* on replacing CarD by CarDNt mutants.** (**A**) Colony color phenotype of the indicated *M. xanthus* strains. 5 µl drops from each liquid culture (OD_550_ ~0.8) were spotted on two CTT plates, grown for 12 hr at 33 °C in the dark, after which one plate continued to be incubated in the dark while the other was exposed to light for the indicated time. (**B**) Reporter P_QRS_::*lacZ* expression (β-Gal activity) measurements for exponentially growing cells of each of the indicated strains in the dark (filled bars) or after 14 h hours under light (unfilled bars).


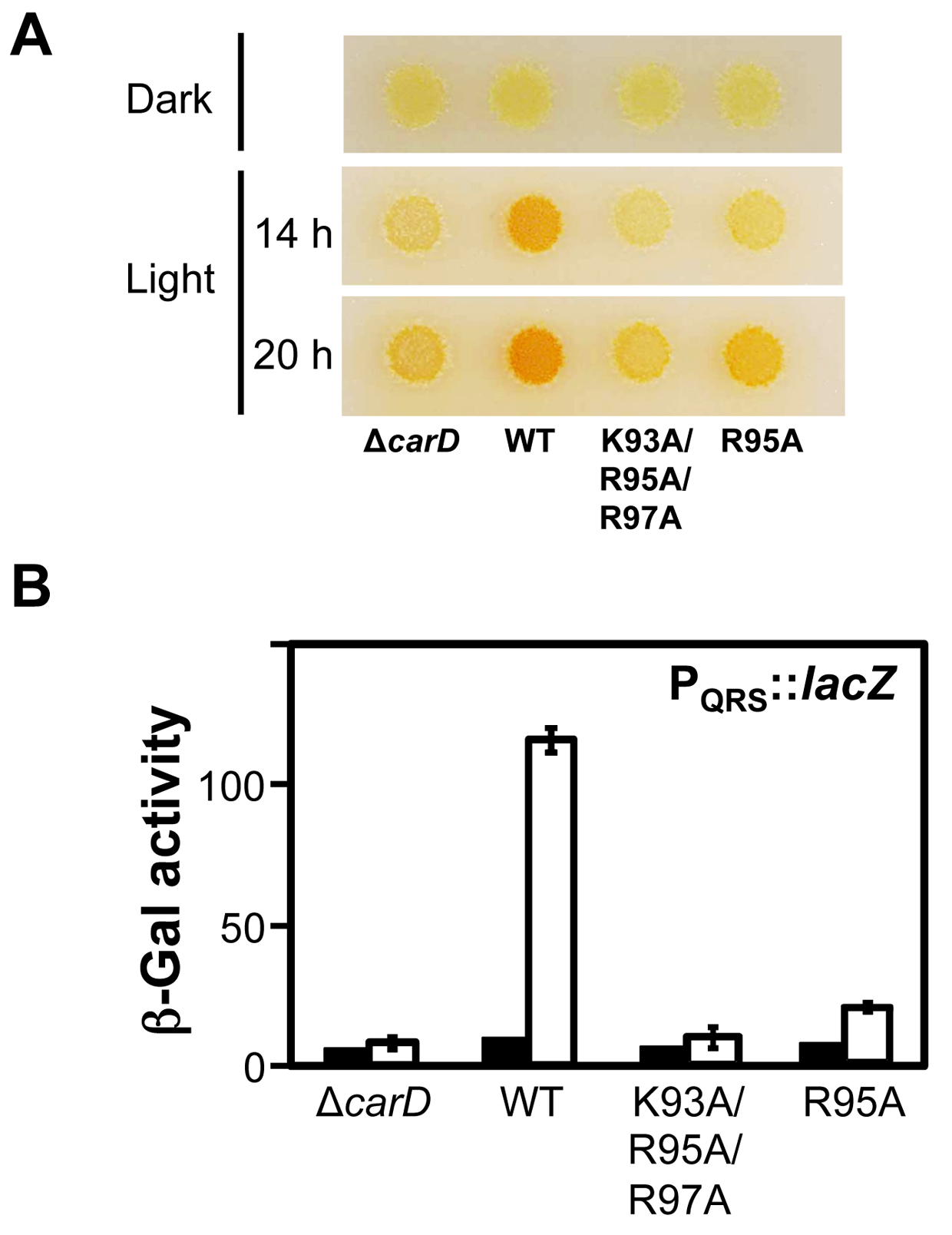


**Figure C. Effect of the R95A mutation on CarD function *in vivo.*** (**A**) Colony color phenotype of *M. xanthus* strains with wild-type *carD* (WT), Δ*carD* and *carD* mutant alleles K93A/R95A/R97A and R95A that were spotted on CTT plates and grown in the dark or under light as described in Figure S2A. (**B**) Reporter P_QRS_::*lacZ* expression (β-Gal activity) measurements for exponentially growing cells of each of the indicated strains in the dark (filled bars) or after 8 hours under light (unfilled bars).

**SI** **REFERENCES**

1. García-Moreno D, Abellón-Ruiz J, García-Heras F, Murillo FJ, Padmanabhan S, Elías-Arnanz M. CdnL, a member of the large CarD-like family of bacterial proteins, is vital for *Myxococcus xanthus* and differs functionally from the global transcriptional regulator CarD. Nucleic Acids Res. 2010; 38: 4586-4598.

2. Abellón-Ruiz J, Bernal-Bernal D, Abellán M, Fontes M, Padmanabhan S, Murillo FJ, et al. The CarD/CarG regulatory complex is required for the action of several members of the large set of *Myxococcus xanthus* extracytoplasmic function sigma factors. Environ Microbiol. 2014; 16: 2475-2490.

3. Cayuela ML, Elías-Arnanz M, Peñalver-Mellado M, Padmanabhan S, Murillo FJ. The *Stigmatella aurantiaca* homolog of *Myxococcus xanthus* high-mobility-group A-type transcription factor CarD: insights into the functional modules of CarD and their distribution in bacteria. J Bacteriol. 2003; 185: 3527-3537.

4. Hodgson DA. Light-induced carotenogenesis in *Myxococcus xanthus*: genetic analysis of the *carR* region. Mol Microbiol. 1993; 7: 471-488.

5. Karimova G, Ullmann A, Ladant D. A bacterial two-hybrid system that exploits a cAMP signaling cascade in *Escherichia coli*. Methods Enzymol. 2000; 328: 59-73.

6. Peñalver-Mellado M, García-Heras F, Padmanabhan S, García-Moreno D, Murillo FJ, Elías-Arnanz M. Recruitment of a novel zinc-bound transcriptional factor by a bacterial HMGA-type protein is required for regulating multiple processes in *Myxococcus xanthus*. Mol Microbiol. 2006; 61: 910-926.
